# A Novel Web-Based Application for Influenza and COVID-19 Outbreak Detection and Response in Residential Aged Care Facilities

**DOI:** 10.2196/37625

**Published:** 2024-06-24

**Authors:** Kai Hsun Hsiao, Emma Quinn, Travers Johnstone, Maria Gomez, Andrew Ingleton, Arun Parasuraman, Zeina Najjar, Leena Gupta

**Affiliations:** 1 Public Health Unit, Sydney Local Health District, Sydney, Australia; 2 School of Public Health, Faculty of Medicine and Health, University of Sydney, Sydney, Australia

**Keywords:** web application, digital health, communicable disease control, outbreak, surveillance, influenza, aged care, aged care homes

## Abstract

The use of innovative digital health technologies in public health is expanding quickly, including the use of these tools in outbreak response. The translation of a digital health innovation into effective public health practice is a complex process requiring diverse enablers across the people, process, and technology domains. This paper describes a novel web-based application that was designed and implemented by a district-level public health authority to assist residential aged care facilities in influenza and COVID-19 outbreak detection and response. It discusses some of the challenges, enablers, and key lessons learned in designing and implementing such a novel application from the perspectives of the public health practitioners (the authors) that undertook this project.

## Introduction

Digital health technologies such as web- and mobile-based applications have proliferated over the last 2 decades [1]. Historically, these applications have been used in communicable disease surveillance and control [1-3]. However, the public health field is beleaguered by tools that are short-lived, duplicative, disconnected, of poor quality, or not suited to user needs [2,4,5]. There is also growing evidence that there are consistent barriers to effective implementation and adoption of digital health technologies [4]. Some of these include change management, stakeholder acceptance, training, supporting infrastructure, sustainable financial and human resources, and governance for quality and safety [1,4]. It is therefore critical for public health practitioners to recognize the broader set of necessary factors and considerations when planning, designing, and implementing innovative digital health interventions.

In 2017, the Sydney Local Health District (SLHD) Public Health Unit (PHU), a district-level public health authority, embarked on a digital health innovation project to design, develop, and implement a web-based application—the Influenza Outbreak Communication, Advice and Reporting (FluCARE) application—to assist staff in residential aged care facilities (RACFs) in the timely detection and response to influenza outbreaks. Early outbreak notification to the PHU has been shown to reduce outbreak duration and impact [6].

The application was launched as a pilot across the district in late 2019. In 2020, in response to the COVID-19 pandemic, rapid adaptations were made to FluCARE to incorporate COVID-19 monitoring, supported by additional procedures, guides, and training modules. FluCARE continues to be used by RACFs across the district. The purpose of this paper is to describe the design, development, and implementation of the FluCARE application, as well as key challenges, enablers, and lessons learned from the perspective of the public health practitioners (the authors) who implemented the application.

## Setting and Context

FluCARE was undertaken in the SLHD, which encompasses a metropolitan area of Sydney, Australia, with a population of 640,000, of which approximately 80,000 (12%) are aged 65 years and older [7]. Within the district, there are 62 RACFs, which vary in size, care levels, and ownership, with over 4700 operational bed places in total [8].

The PHU is responsible for local communicable disease control and outbreak management within the SLHD. This includes assisting RACFs in responding to influenza outbreaks, which remains a high priority for public health due to the potential for significant health impacts on older adult residents [6]. While RACFs are not mandated to report influenza outbreaks to the PHU, it is recommended in national guidelines [9]. The national guidelines also provide definitions for suspected and confirmed influenza cases based on their symptoms and laboratory results, as well as the criteria for declaring a potential or confirmed outbreak. Upon the notification of an influenza outbreak, the PHU assists the RACF by advising on response actions and by monitoring outbreak progress through daily line lists of cases submitted by the RACF.

## Design and Development of FluCARE

**Figure 1.**
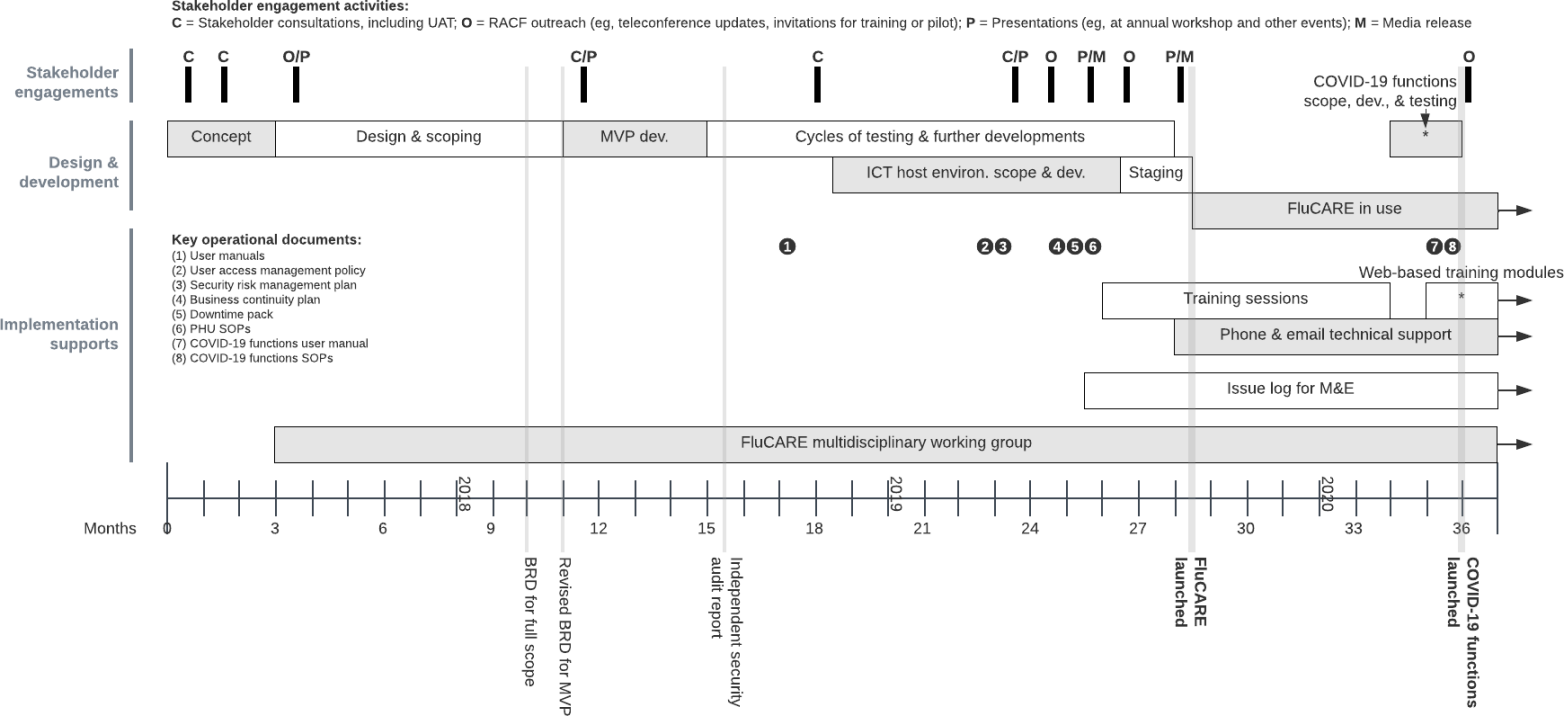
FluCARE project timeline demonstrating the relationships between stakeholder engagement, design and development, and implementation support activities. Staging refers to a prelaunch phase of FluCARE testing within the SLHD hosting environment. *Web-based training modules were offered through the Moodle platform. BRD: business requirements document; dev.: development; environ.: environment; FluCARE: Influenza Outbreak Communication, Advice and Reporting application; ICT: information and communication technology; M&E: monitoring and evaluation; MVP: minimum viable product; RACF: residential aged care facility; SLHD: Sydney Local Health District; SOP: standard operating procedure; UAT: user acceptability testing.

An overview of our application design, development, and implementation activity is shown in Figure 1.

### Conceptual Design

Application scoping and conceptual design occupied a significant portion (8 months) of our project timeline due to the need to ensure that we had the appropriate type and level of functional capability included in the application. A variety of different stakeholder consultation sessions were held with RACF and PHU staff to understand design needs (see Table 1, item 6), and through the establishment and use of a working group, we were able to finalize and agree on a minimum viable product (MVP; see Figure 1).

**Table 1.** Description of the design and technical features of FluCARE^a^ as per the WHO^b^ Mobile Health Evidence Reporting and Assessment (mERA) criteria [10].

Item	mERA criteria or feature	Description
1	Infrastructure	FluCARE is a web-based application. User access requires internet connectivity and a web browser, which are typically available in the PHU^c^ and the RACFs^d^ in the district. The application is optimized for desktop and laptop computers but can also be accessed by tablet and mobile devices. Users also require access to an individual email account and/or mobile phone to receive MFA^e^ codes. While smartphone device ownership in Australia is 91% [11], their use by RACF staff at work may be limited by workplace policies and/or signal strength. The FluCARE application and associated databases were initially hosted on a local server maintained within the SLHD^f^ secure network. At the time of writing this viewpoint, the application was being transitioned into the Microsoft Azure cloud environment.
2	Technology platform	FluCARE is an ASP.NET Core MVC^g^ web application written in the C# language with the Razor View engine and Telerik controls. Sensitive data are encrypted and held in a Microsoft SQL Server database. SendGrid is used for the delivery of email notifications and MFA codes. Amazon SNS^h^ is used for the delivery of SMS MFA codes. FluCARE is hosted on IIS^i^ in a Windows environment.
3	Interoperability	FluCARE is not directly connected to any other local or state health information system. However, relevant reports can be generated and exported as a CSV file, which can be configured for upload to the NSW^j^ Notifiable Conditions Information Management System where PHUs are required to report and document RACF influenza outbreaks.
4	Intervention delivery	RACFs register for and access the FluCARE application via the application web page. The PHU promotes RACF awareness, access, and use of FluCARE via the PHU’s annual workshop on winter preparedness for RACFs, regular email reminders, and occasional update teleconferences.
5	Intervention content	FluCARE content and processes are based on the national CDNA^k^ guidelines for the management of influenza outbreaks in RACFs [9]. Application content was developed, adapted, and reviewed by a multidisciplinary working group.
6	Design and development	End-user engagement and testing were conducted at multiple points throughout the design and development process (see Figure 1): Initial RACF staff consultation via an open forum discussion and structured survey, and key informant interviews with PHU staff on their surveillance needs informed the conceptual design of FluCARE. Preproduction wireframes were presented to PHU and RACF staff (both at an annual winter preparedness workshop and in facility-based meetings) to seek feedback on user-friendliness, aesthetics, and functions prior to MVP^l^ launch (see Figure 1). Postproduction UAT^m^ was conducted with RACF staff focus groups (not reported in detail here).
7	User feedback	UAT conducted with a focus group of RACF staff found the application design to be user-friendly and accessible, the content informative, and the functionalities useful and likely to save time and reduce workload. Most importantly, the UAT found that most RACF staff users would access and use the application on their desktop, rather than on mobile devices at the bedside, which led us to optimizing the application for desktop use.
8	Access of individual participants	Access to the FluCARE application requires a desktop computer or mobile phone and internet connection either at work or home.
9	Cost assessment	Economic evaluations of FluCARE are yet to be conducted.
10	Adoption inputs/program entry	PHU and RACF users are required to complete training on FluCARE via either a 2.5-hour face-to-face training session or a set of web-based modules. User manuals are available within FluCARE. Designated PHU staff provide users with email and telephone support from initial registration to operational use of FluCARE.
11	Limitations for delivery at scale	Application development will be necessary to allow FluCARE to cater for multiple districts or jurisdictions. Currently, further scale-up of FluCARE is limited by our district’s IT infrastructure and human resource capacity to support an expanded number of RACFs and users. Implementation research is needed to inform and adapt strategies to support FluCARE implementation to other LHDs^n^ or PHUs.
12	Contextual adaptability	FluCARE was built as a proof of concept with a focus on influenza outbreaks in RACFs in our district. However, we believe it would be adaptable for other communicable diseases, settings, districts, or jurisdictions. For example, FluCARE was rapidly redeveloped to incorporate COVID-19 outbreak monitoring in RACFs.
13	Replicability	The FluCARE application and supporting processes, such as user manuals, training modules, and standard operating procedures, can be replicated for introduction into new districts or jurisdictions.
14	Data security	Application-level security includes ASP.NET-secured cookie authentication, a strong password policy, MFA for user access, autolocking accounts for excess failed log-ins, and inactivity timeout. Data are secured with 256-bit encryption. System servers are secured by firewalls. Procedural protections include manual verification prior to RACF account approval, user terms and conditions, and a user management policy based on a principle of least privilege.
15	Compliance	FluCARE is compliant with the NSW Health Records and Information Privacy Act 2002 [12] and relevant NSW Health governing standards, that is, the Health Care Records Policy Directive [13] and the Privacy Manual for Health Information [14]. This was verified by an independent security audit. The content, algorithms, and advice in FluCARE align with the national CNDA guidelines for the prevention, control, and public health management of influenza outbreaks in RACFs in Australia [9].
16	Fidelity	FluCARE underwent a pilot feasibility study, which demonstrated high acceptance, utility, and safety for RACF staff in the management of influenza and COVID-19 outbreaks [15].

a FluCARE: Influenza Outbreak Communication, Advice and Reporting.

b WHO: World Health Organization.

c PHU: Public Health Unit.

d RACF: residential aged care facility.

e MFA: multifactor authentication.

f SLHD: Sydney Local Health District.

g MVC: Model-View-Controller.

h SNS: Simple Notification Service.

i IIS: Internet Information Services.

j NSW: New South Wales.

k CDNA: Communicable Diseases Network Australia.

l MVP: minimum viable product.

m UAT: user acceptablility testing.

n LHD: local health district.

Through the RACF stakeholder consultation sessions (see Figure 1), the PHU identified 3 key barriers to effective influenza surveillance and outbreak response and, with the working group, designed a series of features that addressed these barriers, as shown in Table 2 below. The working group membership included clinical and epidemiological staff from within the PHU, geriatric care managers and senior information and communication technology (ICT) staff from across the district, and managers of respiratory diseases from within Health Protection NSW. The group met weekly to advise on the components or features of the application and together produced a comprehensive business requirements document from which only the essential elements for an initial MVP build had to be identified and agreed on (see Figure 1).

**Table 2.** Residential aged care facility (RACF) barriers to influenza outbreak control, and associated key design features of FluCARE^a^ to improve outbreak management.

Key barriers to influenza outbreak control	Key FluCARE application design features
Emailed or faxed line listing	*Web-based line list and interface for data management (see *Figure 2): The interface allows RACF staff to log onto their facility-specific account, add or update line list entries, and directly submit to the PHU^b^ via the application. The PHU can immediately view and manage all RACF line lists.
Complicated case definitions and outbreak criteria	*Real-time outbreak detection algorithms:* FluCARE analyzes the line list data to (1) highlight entries meeting case definitions for influenza-like illness or confirmed influenza and (2) detect when the criteria for a potential or confirmed influenza outbreak are met. *An outbreak action checklist:* Tick-box list of key response actions that the RACF should take based on guidelines.
Need to notify multiple stakeholders of an outbreak in a timely fashion	*Automated stakeholder notifications once the outbreak criteria have been met:* Upon detecting an outbreak situation, FluCARE sends email notifications to: the PHU, including to prompt verification; key RACF staff, such as the facility or infection control manager; and any additional notification recipients, such as residents’ general practitioners, as subscribed by the RACF. *Daily line list report:* Enables the RACFs and the PHU to report on the progress and response to the outbreak to senior management.

a FluCARE: Influenza Outbreak Communication, Advice and Reporting application.

b PHU: Public Health Unit.

**Figure 2.**
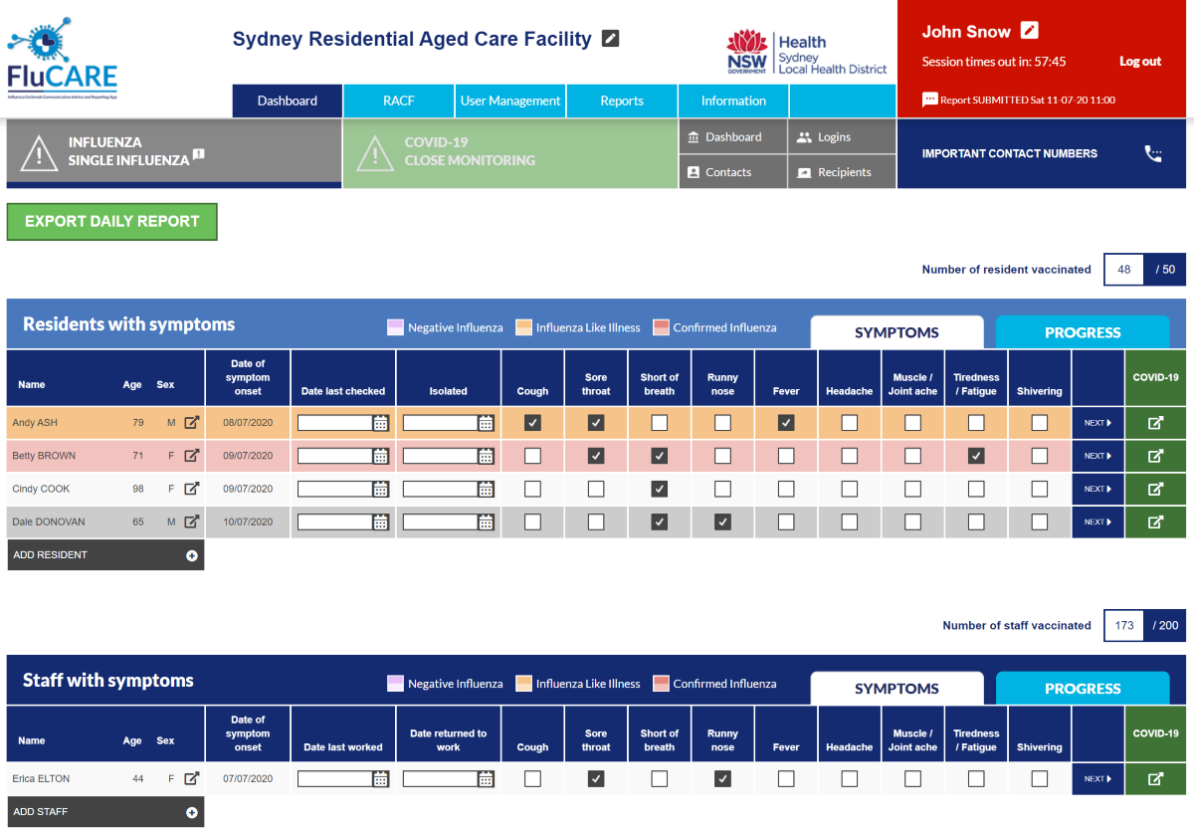
Screenshot of the residential aged care facility (RACF) dashboard and web-based line list interface in the Influenza Outbreak Communication, Advice and Reporting (FluCARE) application. This is the most recent version of FluCARE, with rapid add-on COVID-19 functionalities visible. (Displayed names are fictional). NSW: New South Wales.

### Development

Once the MVP was developed, a comprehensive audit of the application’s content, algorithm, and functions was conducted, as well as external user acceptability testing (UAT) with a focus group of self-volunteered RACF staff (see Figure 1). The UAT was designed as a guided, hands-on training session on FluCARE followed by a structured feedback session with comments obtained via moderated group discussion. This testing identified minor issues and potential improvements, such as changes to interface workflow. The MVP then underwent further cycles of testing and enhancement prior to pilot launch. After the MVP launch, an independent security assessment verified that the technical and architectural design of the application met governing standards (Table 1, item 15 and Figure 1). Further to this audit and to support appropriate local procedures in how FluCARE data were managed, the PHU underwent an internal security risk assessment and developed a risk management plan with our IT services prior to launch. This was particularly important as the application transitioned from the digital developer’s environment to being hosted by the SLHD ICT local servers (see Figure 1).

Even after launch, the FluCARE application underwent further refinements over time in accordance with PHU or RACF feedback. The most significant enhancement was a rapid 6-week development in March 2020 to incorporate COVID-19 monitoring and outbreak detection (see Figure 1). At the time of developing the FluCARE application, there was no standardized and validated way to report the features of a web-based application, so the features of FluCARE have been described as per the World Health Organization checklist on Mobile Health Evidence Reporting and Assessment criteria above (see Multimedia Appendix 1 [10]).

## Implementation Process

**Figure 3.**
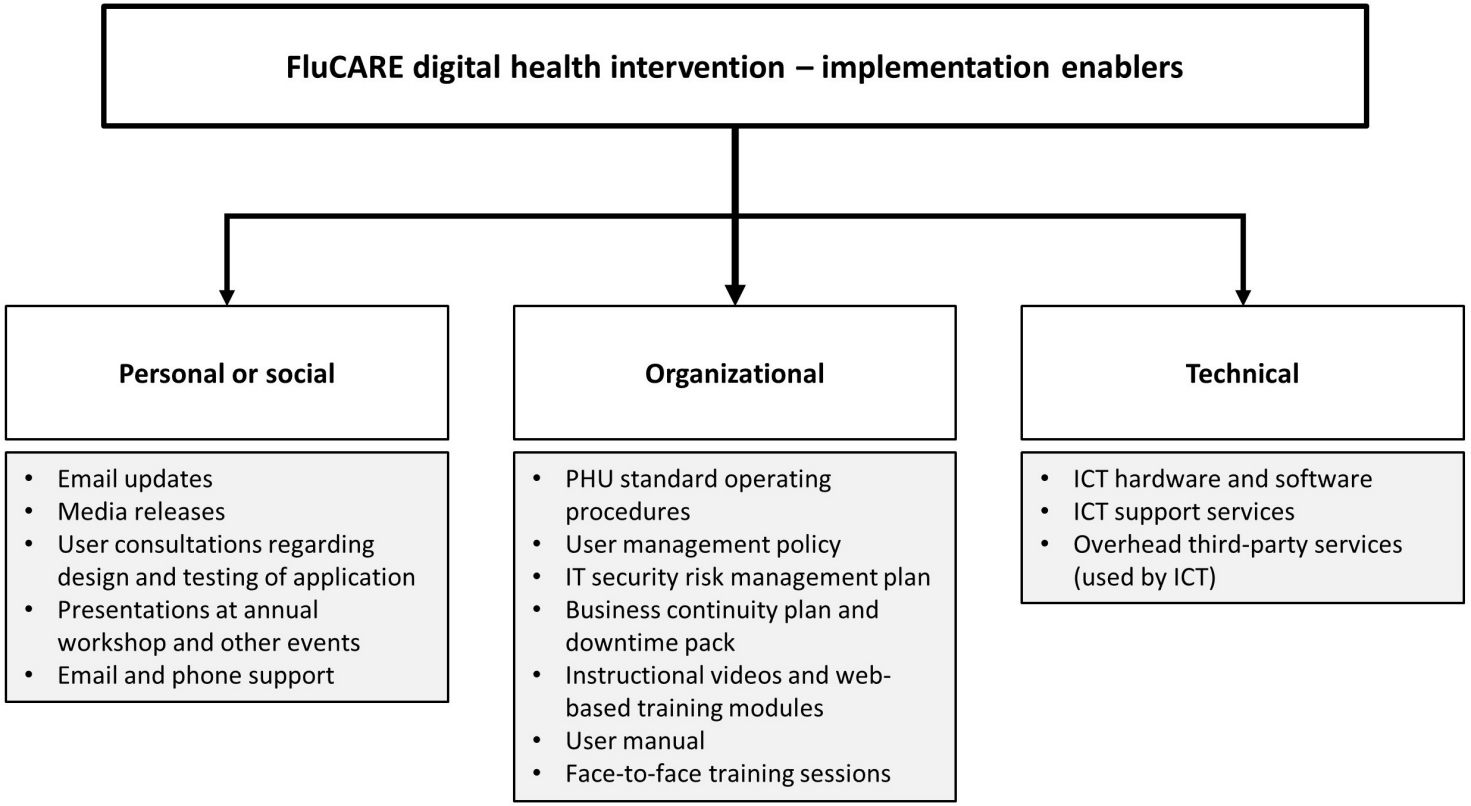
Overview of the FluCARE digital health implementation package as informed by Cresswell and Sheikh [16] and Gagnon et al [17]. FluCARE: Influenza Outbreak Communication, Advice and Reporting application; ICT: information and communication technology; PHU: Public Health Unit.

Preparations for application implementation occurred in parallel to the development process, as illustrated in Figure 1. The authors designed the implementation supports based on 2 reviews of the literature [16,17] focused on the implementation and adoption of health technologies by health care professionals, as shown in Figure 3.

Existing engagements with RACFs, such as the annual winter preparedness workshop, as well as FluCARE-specific outreach, including the various UAT episodes (Table 1, item 6), were used to raise awareness and user buy-in of FluCARE (Figure 3; personal and social enablers). A range of training resources and sessions (described in Table 1, item 10; see Figure 1) were developed by the working group to support PHU and RACF staff onboarding. Operationally, a suite of policies and procedures were developed to govern the use of FluCARE as well as to manage issues typically introduced by digital technologies, such as user management, data security, and downtime contingency plans (see Figure 1, items 1‐8 and Figure 3; organizational enablers).

Internal IT infrastructure and operational support from the SLHD ICT Services were also critical. The SLHD ICT Services built the requisite highly available and secure server environment to host FluCARE and continue to provide services for maintenance and issues resolution (see Figure 3; technical enablers).

For ongoing monitoring, quality improvement, and safety, the PHU established a registry (issue log) to prospectively record technical or operational issues and any improvement suggestions (see Figure 1). These are routinely reviewed by the working group for further updates to the application (technical enablers). Further evaluation of the implementation of FluCARE through a feasibility study has been published [15], and effectiveness studies are planned.

## Discussion

Despite substantial challenges related to the redesign of the application during the COVID-19 response, the main lessons learned across the entire design, development, and implementation time frame of FluCARE (as shown in Figure 1) were the importance of user-based design activity, information security and privacy, and organizational support (such as funding and IT infrastructure).

### User-Related Lesson: User Engagement Is Critical and Should Be Embedded in All Phases of Design, Development, and Implementation

User-centric design is a well-understood principle in IT projects [3,5,18-20]. From the outset, we were mindful that FluCARE’s capacity to improve outbreak outcomes was dependent on its ability to be accepted and adopted by RACF staff. This is typically determined by factors such as an application’s perceived usefulness, reliability, and user-friendliness [5,21]. As such, input from RACF staff was pivotal. Beyond informing the core functions of the application, feedback from RACF staff via the various user engagement and testing episodes embedded in our design and development process (see Figure 1; 0-27 months) was valuable in answering key design questions encountered by the working group and digital developers, for example, whether to optimize the application for desktop versus mobile use. Lastly, while acknowledging the importance of user-based design in the development of a digital health tool, no one design approach is accepted as a gold standard [22]. However, we did not conduct our user-based design activity through a theoretical framework or research process and would have benefited from such a systematic approach.

### Technical Lesson: Data Security Should Be Addressed With a Multifaceted, Risk Management Approach

Data security and information privacy is a central concern that must be thoroughly addressed to ensure user acceptability, and that ethical and professional obligations are met, particularly when dealing with personally identifiable health data [5,23]. Addressing all facets of security appropriately required not only an understanding of the legislation, policies, and procedures in place to secure and manage health data appropriately within New South Wales but also a practical risk management approach in order to mitigate any perceived risks to data breaches. Based on the recommendations from the independent security assessment, the working group developed a user access management policy and security risk management plan to help mitigate any inappropriate access or use of the application and to ensure the security of the personal health information within the application (Figure 1; 15‐27 months). While much of the existing literature focuses on the technical aspects of security, it is important to recognize that data governance strategies, staff training, and incident detection and response plans are also essential for security [24-26]. This multifaceted security approach was applied for FluCARE, as detailed in Table 1 (item 14).

### Organizational Lesson: Organization-Wide Support for Digital Innovation Should Be Fostered and Promoted

Supportive organizational culture and structures for digital health innovation are important enablers for successful design and implementation [5,27]. FluCARE development was undertaken within a forward-thinking organization where innovation in health care IT is a strategic priority [28]. The project received high-level support at initiation through the SLHD executive as well as at the PHU and IT departmental levels, which facilitated supportive resourcing from the IT department, including their de novo build of the required hosting environment for FluCARE. However, we may have potentially benefited from an organization-wide mechanism for pooling institutional experience and sharing resources across different projects, given that a number of other application developments were also occurring in the SLHD over the time period of the design, development, and implementation of FluCARE (see Figure 1). This has potential for leveraging economies of scale, reducing duplicative overhead costs, optimizing interoperability, and improving procedural efficiencies [5,29].

## Conclusion

The translation of an innovative application idea into an effective public health tool is a multifaceted process, which importantly includes early and ongoing user engagement, considered but practical data governance and security measures, organizational support, and dedicated resources and IT infrastructure to support maintenance over time. From our experience, these elements are essential to ensure user-friendly and security-conscious design as well as successful application implementation and uptake.

## Supplementary material

10.2196/37625 Multimedia Appendix 1 Checklist for Mobile Health (mHealth) Evidence Reporting and Assessment (mERA) guidelines, including mHealth essential criteria.

## Abbreviations

FluCARE: Influenza Outbreak Communication, Advice and Reporting
ICT: information and communication technology
MVP: minimum viable product
PHU: Public Health Unit
RACF: residential aged care facility
SLHD: Sydney Local Health District
UAT: user acceptability testing
